# *Brucella melitensis* Rev.1 vaccination generates a higher shedding risk of the vaccine strain in Alpine ibex (*Capra ibex*) compared to the domestic goat (*Capra hircus*)

**DOI:** 10.1186/s13567-019-0717-0

**Published:** 2019-11-27

**Authors:** Claire Ponsart, Mickaël Riou, Yann Locatelli, Isabelle Jacques, Alain Fadeau, Maryne Jay, Roland Simon, Ludivine Perrot, Luca Freddi, Sylvain Breton, Thierry Chaumeil, Barbara Blanc, Katia Ortiz, Colin Vion, Damien Rioult, Erwan Quéméré, Pierre Sarradin, Jean-Yves Chollet, Bruno Garin-Bastuji, Sophie Rossi

**Affiliations:** 1 EU/OIE/FAO & National Reference Laboratory for Animal Brucellosis, Animal Health Laboratory, ANSES/Paris-Est University, 94706 Maisons-Alfort, France; 2 UE-1277 Plateforme d’Infectiologie expérimentale (PFIE), INRA Centre Val de Loire, 37380 Nouzilly, France; 3 Réserve Zoologique de la Haute Touche, Muséum National d’Histoire Naturelle (MNHN), 36290 Obterre, France; 4 Département Génie Biologique, Institut Universitaire Technologique (IUT), Université de Tours, 29 Rue du Pont Volant, 37082 Tours Cedex 2, France; 5 UMR-1282 Infectiologie et Santé Publique (ISP), INRA Centre Val de Loire – Université de Tours, 37380 Nouzilly, France; 6 Laboratoire de Touraine, Conseil départemental d’Indre-et-Loire, B.P. 67357, 37073 Tours Cedex 02, France; 7 Université de Reims Champagne-Ardenne, 9 Boulevard de la Paix, 51100 Reims, France; 8 Unité Comportement et Écologie de la Faune Sauvage (CEFS), INRA, 24 chemin de Borde-Rouge-Auzeville CS 52627, 31326 Castanet-Tolosan Cedex, France; 9 Unité sanitaire de la Faune, Direction de la Recherche et de l’Expertise (DRE), Office National de la Chasse et de la Faune Sauvage (ONCFS), 5 rue de Saint-Thibaud, Saint-Benoît, 78610 Auffargis, France; 10 European and International Affairs Department, ANSES, 94701 Maisons-Alfort, France

## Abstract

Epidemiological investigations implemented in wild and domestic ruminants evidenced a reservoir for *Brucella* in *Capra ibex* in the French Alps. Vaccination was considered as a possible way to control *Brucella* infection in this wildlife population. Twelve ibexes and twelve goats were allocated into four groups housed separately, each including six males or six non-pregnant females. Four to five animals were vaccinated and one or two animals were contact animals. Half of the animals were necropsied 45 days post-vaccination (pv), and the remaining ones at 90 days pv. Additional samples were collected 20 and 68 days pv to explore bacterial distribution in organs and humoral immunity. Neither clinical signs nor *Brucella*-specific lesions were observed and all vaccinated animals seroconverted. *Brucella* distribution and antibody profiles were highly contrasted between both species. Proportion of infected samples was significantly higher in ibex compared to goats and decreased between 45 and 90 days pv. Two male ibex presented urogenital excretion at 20 or 45 days pv. The bacterial load was higher 45 days in ibexes compared to goats, whereas it remained moderate to low 90 days pv in both species with large variability between animals. In this experiment, differences between species remained the main source of variation, with low impact of other individual factors. To conclude, multiplicative and shedding capacity of Rev.1 was much higher in ibex compared to goats within 90 days. These results provide initial information on the potential use *in natura* of a commercial vaccine.

## Introduction

*Brucella melitensis* is a Gram-negative facultative intracellular bacterium responsible for brucellosis in small ruminants, a widespread zoonosis in many sheep- and goat-raising countries worldwide [1–4].

Brucellosis eradication in small ruminants has been achieved in most of European Union (EU) countries through the implementation of long-term management programs combining vaccination with serological testing and culling [5]. Until recently, terrestrial wildlife had not been considered as a significant reservoir [6]. In France, no brucellosis cases have been reported in domestic ruminants since 2003 [7]. However, *B. melitensis* biovar 3 infection has been identified since 2012 in Alpine ibexes (*Capra ibex*) in the Bargy Mountains (Haute Savoie, France), after a local outbreak on a dairy cattle farm and two human cases declared in 2011 [8–10]. The restriction of brucellosis to the Bargy area indicates a localized outbreak in wildlife. Until recently, Alpine ibexes had been considered to be epidemiological dead-ends for *B. melitensis* [6], but the high prevalence observed in the Bargy area (38%) suggested the presence of an unexpected wildlife reservoir [11]. Focused culling of seropositive or ill ibexes and mass culling have been implemented since 2013, which reduced the population by half [12], raising the question of the social acceptability of conducting mass culling in a protected species [11]. Moreover, this management strategy did not result in a significant reduction in seroprevalence [11, 12].

A scientific expert appraisal suggested that vaccination with the *B. melitensis* Rev.1 strain of ibex could be considered for better control of this wildlife reservoir [13, 14]. The Rev.1 vaccine, a stable live *B. melitensis* attenuated strain [15, 16], administered by the conjunctival route at standard doses is well known to induce good protection in sheep and goats against *B. melitensis*-related abortion [17, 18]. The main risks of vaccination are potential abortion in female animals vaccinated during pregnancy and possible Rev.1 genital or milk excretion following vaccination [19–23]. Despite the phylogenetic closeness of ibex and domestic goats (*Capra hircus*) [24], experts have highlighted the importance of confirming vaccine safety in Alpine ibex before its application *in natura* [13, 25], taking into account the other potential negative impacts of a live *Brucella* vaccine reported in other wildlife species [26–30], and potential interference with local monitoring and management programs [25]. It was therefore decided to design a study aimed at checking the innocuousness of Rev.1 in non-pregnant sexually mature ibexes that are the most common captured/sampled age class (juveniles being, on the contrary, rarely captured) [12].

Because of ethical, regulatory, logistic and practical reasons, a virulent challenge in pregnant Alpine ibex requiring a biosafety level 3 (BSL-3) facility adapted to this wildlife species could not be considered [25].

Therefore, the objective of the present study was to assess the innocuousness of the Rev.1 conjunctival vaccine in non-pregnant sexually mature Alpine ibexes compared to domestic goats, and the subsequent risk of shedding and transmission to unvaccinated control animals. The hypothesis tested here was that sexually mature ibexes and goats have a comparable ability to control the vaccine.

## Materials and methods

All experiments were conducted in accordance with EU guidelines and French regulations (Directive 2010/63/EU, 2010; French Rural Code, 2018; French Decree No. 2013-118, 2013, [31]). All experimental procedures were evaluated and approved by the Ministry of Higher Education and Research (Notifications: APAFIS#7643-2016112111336721 v4 and APAFlS#7913-2016112911444302 v3). Procedures concerning goats were evaluated by the Ethics Committee of the Val de Loire (CEEA VdL, committee No. 19, APAFIS#7643) and took place at the INRA Experimental Infection Platform [32], whereas procedures for ibex were evaluated by the Cuvier Ethics Committee (CEEA Cuvier, Committee No. 68, APAFIS#7913) and took place at the *Réserve Zoologique de la Haute Touche* (RZHT, Obterre, France).

### Selection of animals

A major difficulty in the present study was to pair goats and ibexes regarding their sexual maturity and health status, which is known to impact individual susceptibility to *Brucella* rather than age, since sexual maturity in ibex is much later (2 to 5 years) compared to the domestic goat. Goats, on the contrary, are seldom raised for more than 4–5 years (roughly 4–6 months for bucks and 6–18 months for goats [33]). Six male and six non-pregnant female ibexes were recruited from three zoological parks (Parc des Angles, Domaine de Pescheray and RZHT, France). For the experiment, males and females were housed separately at the RZHT in two groups in a 170 m^2^ facility specifically adapted to their welfare. Animal age ranged between 2.5 and 5 years (Table 1, [34, 35]).

**Table 1:** **Individual characteristics of the 12** ***Capra ibex*** **and 12** ***Capra hircus*** **included in the study and applied vaccine treatment**

Species	Group	Housing	Birth date	Identifier	Sex	Age (years)	Treatment	Day of necropsy
*Capra ibex*	A	Haute Touche	01/05/2013	1890	M	3–5	Vaccinated	D45
			01/05/2013	2000	M	3–5	Vaccinated	D90
			01/05/2012	1895	M	3–5	Control	D90
			01/05/2012	1839	M	3–5	Vaccinated	D45
			01/05/2014	1828	M	< 3	Vaccinated	D45
			01/05/2014	3094	M	< 3	Vaccinated	D90
*Capra ibex*	B	Haute Touche	01/05/2012	1926	F	3–5	Control	D90
			01/05/2012	1920	F	< 3	Vaccinated	D45
			01/05/2012	1933	F	3–5	Vaccinated	D45
			01/05/2013	2393	F	< 3	Vaccinated	D90
			01/05/2014	2349	F	< 3	Control	D45
			31/05/2002	7462	F	> 5	Vaccinated	D90
*Capra hircus*	C	PFIE	31/08/2015	62 107	M	< 3	Vaccinated	D90
			04/02/2016	16 142	M	< 3	Vaccinated	D45
			16/02/2016	06 145	M	< 3	Vaccinated	D45
			14/02/2016	61 003	M	< 3	Vaccinated	D45
			15/02/2016	61 275	M	< 3	Vaccinated	D90
			03/12/2012	13 101	M	3–5	Control	D90
*Capra hircus*	D	PFIE	30/08/2010	10 139*	F	> 5	Vaccinated	19/02/2017^a^
			26/08/2011	20 055*	F	> 5	Vaccinated	27/03/2017^a^
			07/09/2012	30 313*	F	3–5	Vaccinated	12/02/2017^a^
			31/08/2013	40 176	F	3–5	Vaccinated	D90
			02/09/2014	50 105	F	< 3	Vaccinated	D90
			27/08/2011	20 072	F	> 5	Control	D90

^a^Death of 3 goats occurred during the experiment, only goat 20 055 was included in the analysis.

*Corresponding to the number of death of 3 goats.

Twelve adult Alpine goats (six non-pregnant females and six males) were obtained from the INRA animal facility in Magneraud (France) and the company CAPGENES (CAPGENES, Agropole, Mignaloux-Beauvoir, France) (Table 1). During the trial, all animals were housed in the animal facilities, biosafety level 1 sheepfold animal facility at the INRA PFIE (INRA Centre de Recherche Val de Loire, Nouzilly, France).

### Vaccination and sampling

**Figure 1:**
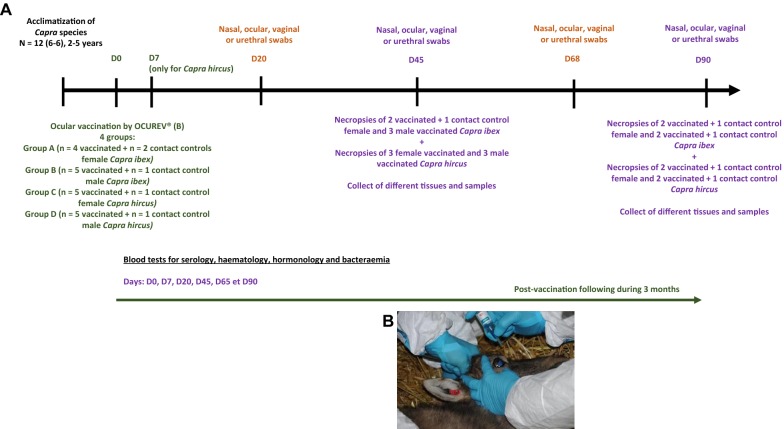
**Experimental design of vaccination with** ***Brucella melitensis*** **Rev.1 strain of** ***Capra ibex*** **and** ***C. hircus.*** **A** Experimental design of ocular vaccination in *Capra ibex* and *hircus* and **B** inoculation of OCUREV^®^ vaccine by ocular pathway at 1 × 10^9^ CFU/drop.

The experiment (Figure 1) consisted in the conjunctival vaccination with Rev.1 vaccine (Ocurev^®^, CZ Veterinaria, Spain; 1–2 × 10^9^ CFU in 35 µL/doses) of 10 goats (divided into two groups: five females and five males) and 9 ibex (divided in two groups: four females and five males; the 5th female ibex was not vaccinated and kept as a contact animal, as diarrhea symptoms were observed on the day of vaccination). At the time of the vaccination, vaccinated animals and contacts were separated from each other in captivity to avoid iatrogenic interference. The animals included in the experiment were neither pregnant nor lactating. For both species, one animal of each sex was used as an unvaccinated contact control, except for the female ibex (two contact controls). Vaccination was systematically performed according to the recommendations of the manufacturer for sheep and goats in the right eyelid. Within each batch, half of the animals (2 individuals vaccinated and 1 control) were euthanized at 45 days pv and the remaining animals at 90 days after anesthesia induction by ketamine/xylazine mixture (Imalgene^®^ 1000, Merial, France, 10 mg/kg and Rompun^®^, Bayer Healthcare, France, 2 mg/kg administered IM). Euthanasia took place on anesthetized animals by IV injection of Euthasol^®^ (Euthasol^®^Vet 40%, LE VET, Netherlands, 1 mL/5 kg) for ibex and Dolethal^®^ (Vetoquinol, France, 250 mL, 50 mg/kg) for goats. All animals were then necropsied.

Serological, bacterial, hematological and hormonal follow-ups were performed at 0, 20, 45, 68, and 90 days pv with an additional blood sample at 7 days pv for goats. In parallel, ocular, nasal and vaginal or urethral swabs were collected for culture purposes (Table 2). Blood samples were collected by jugular venipuncture from all animals. They were prepared for bacteriological culture and assessment of serological response. All animals were blood-tested to check the seroconversion of vaccinated animals and potential exposure to the vaccine strain of unvaccinated contact animals. Vaginal and urethral swabs were performed in females and males, respectively. Ocular and nasal swabs were collected from the inoculated head-side in vaccinated animals and from both sides in contact animals (Table 2). Swabs were prepared for bacteriological culture.

**Table 2:** **Swab and blood collection on days 0, 20, 45, 68 and 90, and urine and tissue collection at necropsy on day 45 or 90 after conjunctival vaccination on day 0 with the** ***B. melitensis*** **Rev.1 strain**

	Vaccinated animals	Contact animals
Collection during post-vaccination kinetics	Ocular and nasal swabs	Ocular and nasal swabs
	From the inoculated head-side (1 swab)	From both head-sides (2 swabs)
	Vaginal and urethral swabs/blood	Vaginal and urethral swabs/blood
Collection at necropsy	Head lymph nodes	Head lymph nodes
	From the inoculated head-side	From both head-sides and pooled
	Iliac, supramammary and inguinal lymph nodes from both sides and pooled	Iliac, supramammary and inguinal lymph nodes from both sides and pooled
	Spleen	Spleen
	Urine aspirated from bladder	Urine aspirated from bladder

As environmental samples, five manure samples per pen were also collected on day 68 pv in both locations (female and male pens).

### Necropsy, tissue collection and preparation

**Figure 2:**
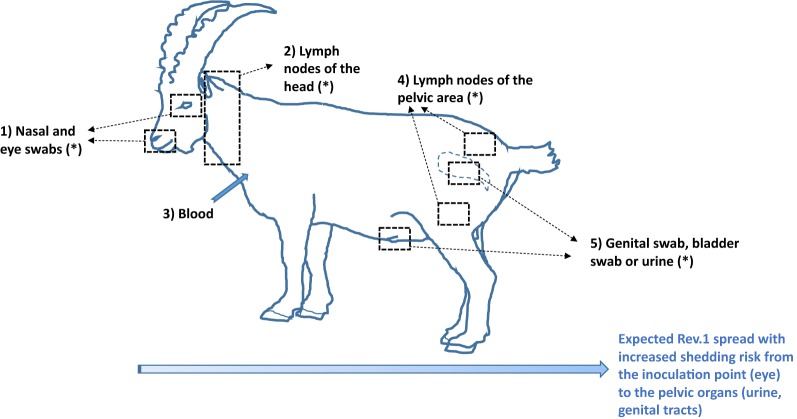
**Organ/swab classification according to localization and shedding potential in both goats and Alpine ibexes.** (1) lymph nodes from the head (local multiplication), (2) nasal and ocular swabs (low shedding potential), (3) blood, (4) lymph nodes from the pelvic area (urogenital multiplication and potential shedding), (5) genital or bladder swabs (high shedding potential).

Necropsies, in a BSL-3 necropsy room, were performed at the PFIE. Urine was collected during necropsy (Table 2). Tissues removed aseptically included parotid, retropharyngeal, sub-maxillary (head lymph nodes), uterus (female) or testes (male), supra-mammary (female) or inguinal (male) and iliac lymph nodes (pelvic organs), pre-scapular lymph nodes, and spleen (Figures 1 and 2). Tissue samples were prepared according to OIE requirements [17] (Table 1). Other specimens were kept frozen at −80 °C.

### Control of the Rev.1 vaccine batch

Concentrations of bacteria in the Ocurev^®^ vaccine batch (number 164164) was previously controlled by the French National Reference Laboratory (NRL) for brucellosis by standard plate counts in accordance with OIE requirements [17] and approved before use. Moreover, one Ocurev^®^ vaccine vial used for goat vaccination was controlled for purity and colonial morphology after conjunctival instillation, according to the same procedure.

### Zootechnical monitoring and behavior

Animal welfare was monitored twice per day by assessing and scoring the following four general categories: (a) behavior, (b) thermal comfort, (c) feeding, and (d) body condition. An endpoint to the experimental procedure was planned according to scoring in case major clinical signs of pain and distress would have been observed: hyperthermia (> 41 °C), prostration, anorexia, diarrhea and/or vomiting, significant weight loss, tissue necrosis, biting.

### Blood cell analyses

Blood cells were counted with a MS9-5 Hematology Counter^®^ (digital automatic hematology analyzer, Melet Schloesing Laboratories, France) and analyzed to characterize total white blood cells, red blood cells, and platelets [36, 37].

### Bacteriological cultures

#### Specimen preparation

Tissue samples and swabs were transferred to the bacteriology laboratory within 2 h following necropsies and then directly handled as following: tissue samples were mixed in a tissue grinder with sterile PBS (two-fold dilution), before being inoculated onto solid media. Each swab was rehydrated in PBS, before being streaked directly onto two plates, then incubated at 37 °C. Whole blood was centrifuged (600 *g*, 15 min). The concentrate was diluted 1:6 in a trypticase soy broth with 5% vol./vol. equine serum. Broths were incubated in a 5% CO_2_ added-atmosphere at 37 °C. Subcultures were initiated every 3 days using Farrell’s solid medium (0.2 mL of broth/dish) until 35 days.

Manure samples were cut and diluted 1:2 in sterile PBS, then incubated overnight at 37 °C, before DNA extraction for molecular detection [38] and culture for bacterial detection. For this particular matrix, the lower limit of detection was determined from manure samples spiked with calibrated broth suspensions of *B. melitensis* biovar 3 (Ether-ATCC 23458).

#### Culture protocol

Culture of blood, swabs and tissue samples was performed on selective modified Farrell’s medium formulated from non-selective solid agar medium and *Brucella* selective supplement (Blood Agar Base No. 2, OXOID CM0271, supplemented with modified *Brucella* Selective Supplement, OXOID SR0209 and 5% vol./vol. equine serum). Each specimen (0.2 mL of prepared specimen) was incubated on 4 plates at 37 °C. The plates were checked for up to 10 days for the presence of bacterial growth; strains were then isolated and identified using a combination of growth characteristics and bacteriological methods [17, 39]. Molecular identification included real-time PCR and Bruce-ladder methods [38, 40].

### Serological analyses

Blood samples were processed according to standard procedures in a BSL-3 laboratory. Whole blood was centrifuged (600 *g*, 15 min). Sera were examined for the presence of smooth *Brucella* antibodies using RBT (Pourquier^®^ Rose Bengal Ag, IDEXX) and CFT (Pourquier^®^ CFT Brucellosis Ag, IDEXX) antigens. The lowest detection limits for CFT assays were determined and expressed as CFT titers (ICFTU/mL). Both antigens were standardized against the OIE International Standard Serum (OIEISS, APHA, Weybridge, UK) and the test performed according to OIE and EU requirements (any visible agglutination reaction was considered positive for RBT; positivity threshold of 20 ICFTU/mL for CFT). RBT results were expressed as a score ranging from 0 (no visible agglutination) to 4 (complete agglutination).

### Statistical analyses

Serological results from vaccinated animals were analyzed according to three criteria: (i) the score of agglutination with the RBT, from 0 (no agglutination) to 4 (complete agglutination); (ii) the CFT titer (ICFTU/mL) corresponding to the end-point of the reaction; and (iii) the individual serological status combining RBT and CFT results: seropositive (positive results with RBT and CFT) or seronegative (negative results with RBT or CFT). To account for the cluster effect of each individual, a mixed model was used, the identity of each individual being used as a random effect [41]. The fixed effects accounted for in serology were the species (ibex, goat), the time of sampling (20, 45, 68, 90 days pv), and the presence of bacteremia on day 20 pv (yes/no). Sex of animals was tested as fixed effect but was not kept in the final model as it didn’t improve significantly the model fitness.

We analyzed bacteriological results from vaccinated animals according to different “innocuousness criteria” accounting for Rev.1 dissemination and the individual ability to contain the live-vaccine: (i) an indicator of Rev.1 strain dissemination among vaccinated individuals, by considering the proportion of organs positive to culture, (ii) an indicator of vaccine strain shedding potential among vaccinated individuals, by considering the proportion of culture-positive organs from the pelvic area; and (iii) the bacterial burden among infected organs or swabs. We analyzed hematological results from vaccinated or non-vaccinated animals according to different “innocuousness criteria” after Rev.1 vaccination in function of time, sex and genders, using GraphPad Prism software version 5.0 (non-parametric tests: Kruskall-Wallis, Mann–Whitney tests, GraphPad^®^, San Diego, Ca, USA).

The two first variables were defined as the proportion of culture-positive organs/swabs/blood sampled on each vaccinated individual and at each sampling time, either at the whole animal level (i) or only the pelvic organs. (ii) Animals excluded from the study before the 20th day post-vaccination (i.e., two dead female goats) were not included in these analyses. To account for the cluster effect of each individual, a logistic mixed model was used, the identity of each individual being used as a random effect [42]. The fixed effects accounted for were species, gender, time of sampling (20, 45, 68, 90 days pv), and type of sampling event (in vivo sampling, necropsy). The third variable was defined as the total number of colonies counted among the culture-positive organs or swabs. (iii) This variable was log-transformed and modelled according to a Gaussian mixed model (random effect). This analysis focused on necropsy samples only (i.e. days 45 or 90 pv). The number of plates was variable among organs (from 1 to 4), mainly due to varying organ/swab nature or size; the number of plates was thus introduced as a model offset. The fixed effects accounted for were species, gender, time of necropsy, and type of organ (Figure 2).

Model selection was based on the Akaike Information Criterion (AIC, [43]). Statistical analyses were performed using R software version 3.4.2 (the R foundation for statistical computing, 2017), and the lme4 [44] and MuMIn packages [45].

## Results

### Vaccine control testing

The control of the Rev.1 vaccine strain performed post-vaccination showed that the strain was pure and smooth. Actual concentration measured by viable counts on TSA-YE medium, 1.6 × 10^9^ CFU/dose, was consistent with that of the manufacturer (1 to 2 × 10^9^ CFU/dose).

### Zootechnical monitoring and behavior

#### Ibexes

No clinical signs were observed during the acclimatization phase. The experimental protocol therefore focused on nine vaccinated animals (five males and four females) and three contact controls (one male and two females). During the experiment, clinical follow-up of the animals showed episodic diarrhea (a gastro-intestinal food supplement of the Phoscargil^®^ type (San’Elevage, Changé, France) was administered to all the animals); one diarrheic female ibex was not vaccinated for this reason and included as a control. No major hyperthermia, polypnea, tachycardia, prostration, anorexia, weight loss or complaints were observed in the animals. Furthermore, in both live animals and at autopsy, no gross lesions suggestive of brucellosis infection (such as described by Freycon et al. and Lambert et al. [46, 47]) were observed in vaccinated or contact animals, particularly in the genitalia, udder or joints. No loss of appetite or body condition, trauma, or aggressive behavior were observed, despite close contact between individuals of the same batch over an extended period of 3 months.

#### Goats

No clinical signs were observed during the acclimatization phase nor during vaccination of the goats, except for a buck that had strong nasal discharge on day 5 pv, which then resolved without treatment. Nevertheless, two vaccinated female goats died without clinical signs on day 6 and day 13 pv, respectively probably in connection with other concomitant diseases and poor body condition of the latter. A third vaccinated goat showed post-vaccination mastitis on day 42 and was euthanized on day 43 for animal welfare reasons (exceeded endpoints).

### Total and differential white blood cell counts in the blood

**Figure 3:**
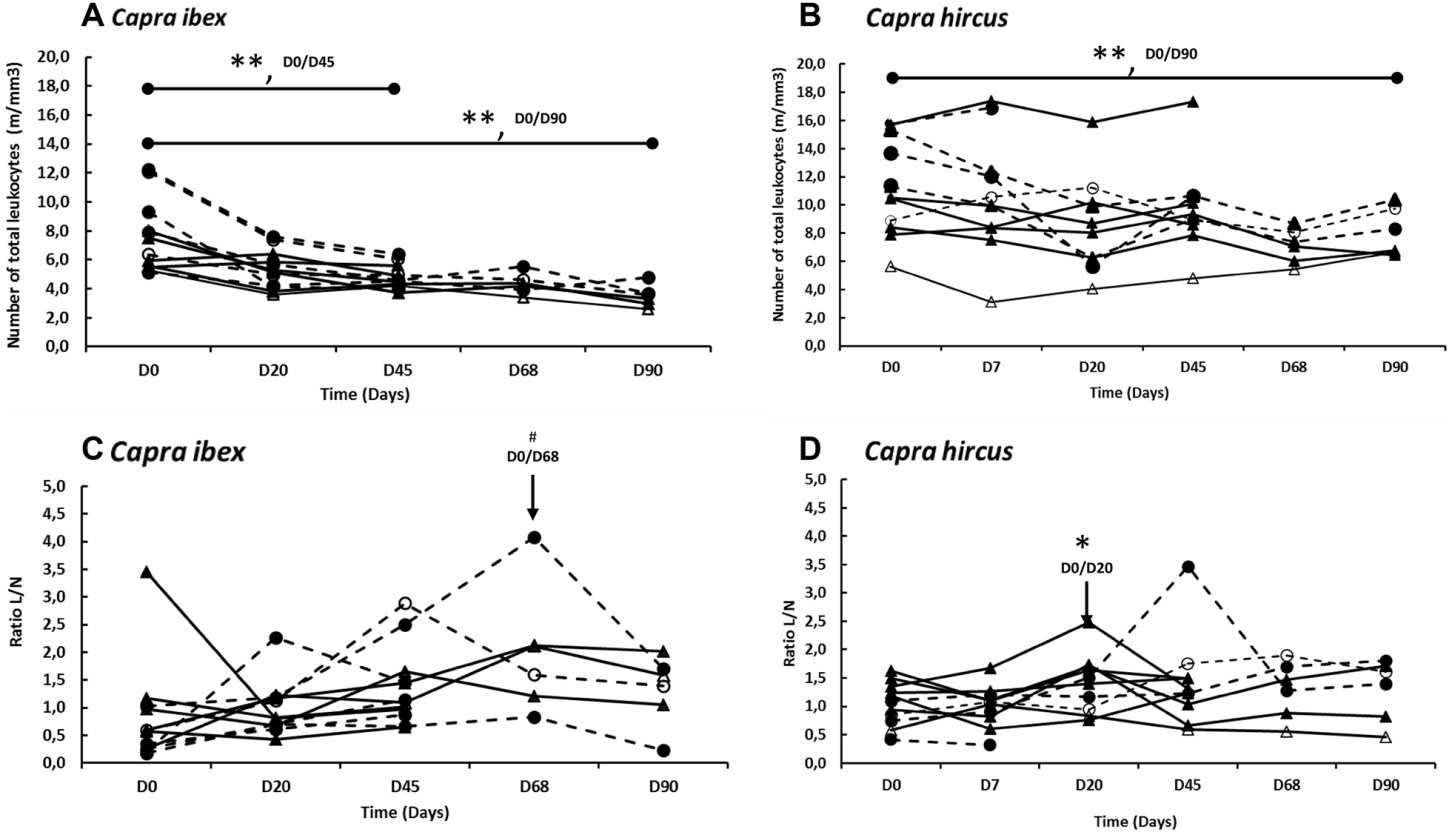
**Evolution of total leucocyte and lymphocyte/neutrophil ratios (L/N) in** ***Capra ib*ex and** ***C. hircus***. Blood was collected on days 0, 7 (only for *C. hircus*), 20, 45, 68 and 90 after conjunctival vaccination on day 0 with *Brucella melitensis* Rev.1 strain. **A**, **C** *Capra ib*ex and **B**, **D** *C. hircus*. Vaccinated males and females (black triangles, continuous lines and black circles-dotted lines, respectively), compared with control animals (**A**–**D**), males and females (empty triangles, continuous lines and empty circles-dotted lines respectively). **P < 0.05; *P = 0.02; ^#^*P* = 0.04.

In addition to significant individual variability, blood counts varied with treatment (vaccination/contact), post-vaccination stage, species, and sex of animals. There was no sex effect on hematological parameters, but a significant effect of species was observed especially for total number of white cells (*P* < 0.05). Following vaccination, a significant decrease in the concentration of white blood cells was observed in both vaccine species on D45 and D90 (*P* < 0.05; Figures 3A and B). No significant decrease during follow-up kinetics was observed in unvaccinated animals. However, the difference between species remained significant throughout the experimental period (*P* < 0.05).

A higher initial lymphocyte to neutrophil (L/N) ratio was observed in ibex females in comparison with female goats (Figure 3C), but this was not significant. In vaccinated ibexes, a gradual increase of the L/N ratio was observed between D0 and D68 and was only significant on D68 compared to D0 (in favor of lymphocytes, *P* = 0.04), corresponding to the multiplication of antibody-producing cells (Figures 3C and D). In vaccinated goats, a more rapid gradual increase of the L/N ratio was observed between D0 and D20 and was only significant on D20 versus D0 (*P* = 0.02). This response occurred later and with a higher intensity in ibexes compared to goats.

### Serological response

**Figure 4:**
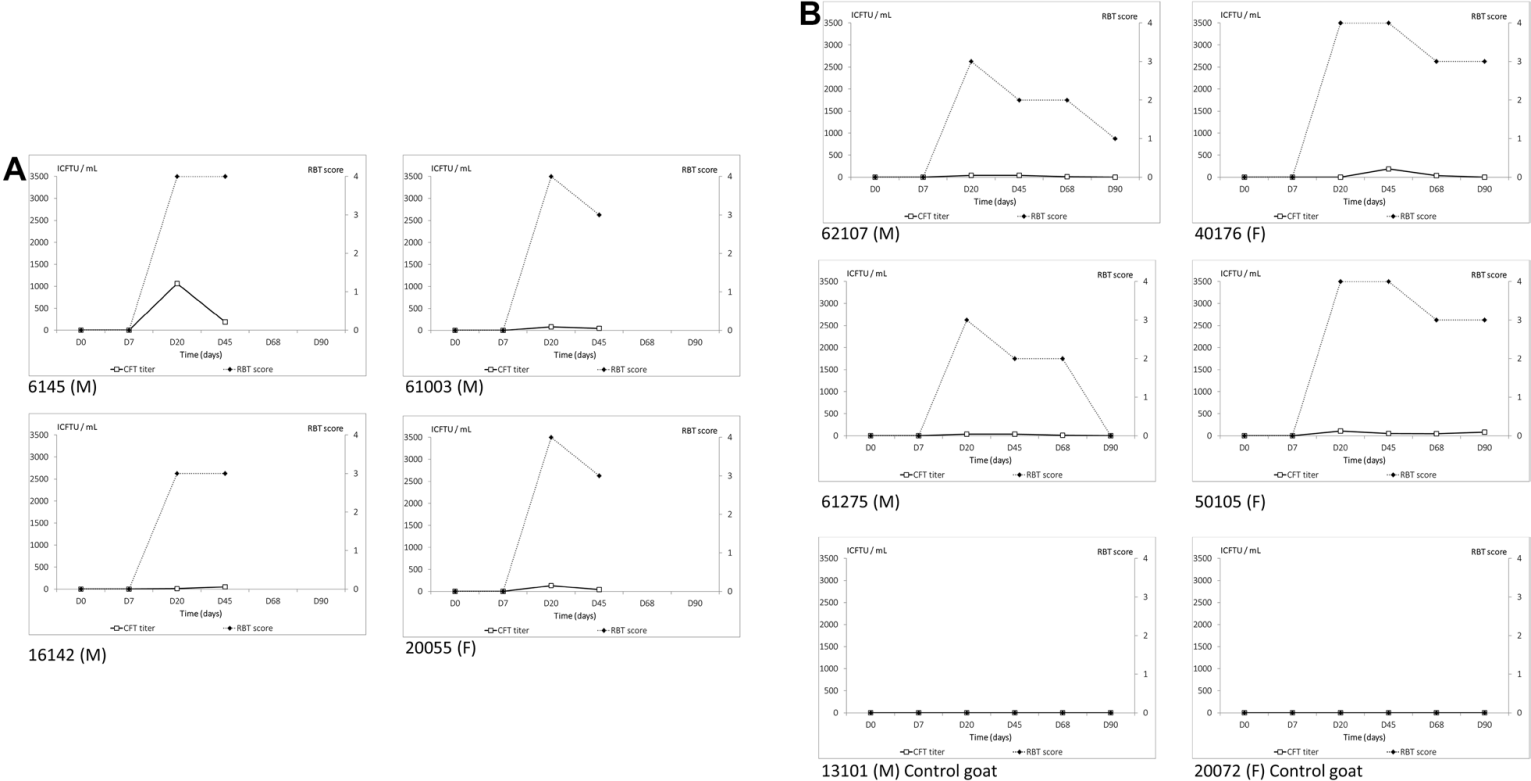
**Evolution of CFT titers (ICFTU/mL) and RBT scores in** ***Capra hircus***. **A** Vaccinated goats, necropsied 45 days post-vaccination, and **B** vaccinated and control goats both necropsied 90 days post-vaccination. Titers and scores were obtained on days 0, 20, 45, 68 and 90 after conjunctival vaccination on day 0 with *B. melitensis* Rev.1 strain, in both males (M) and females (F) (squares continuous lines for CFT titers and respectively diamonds in dotted lines for RBT score).

**Figure 5:**
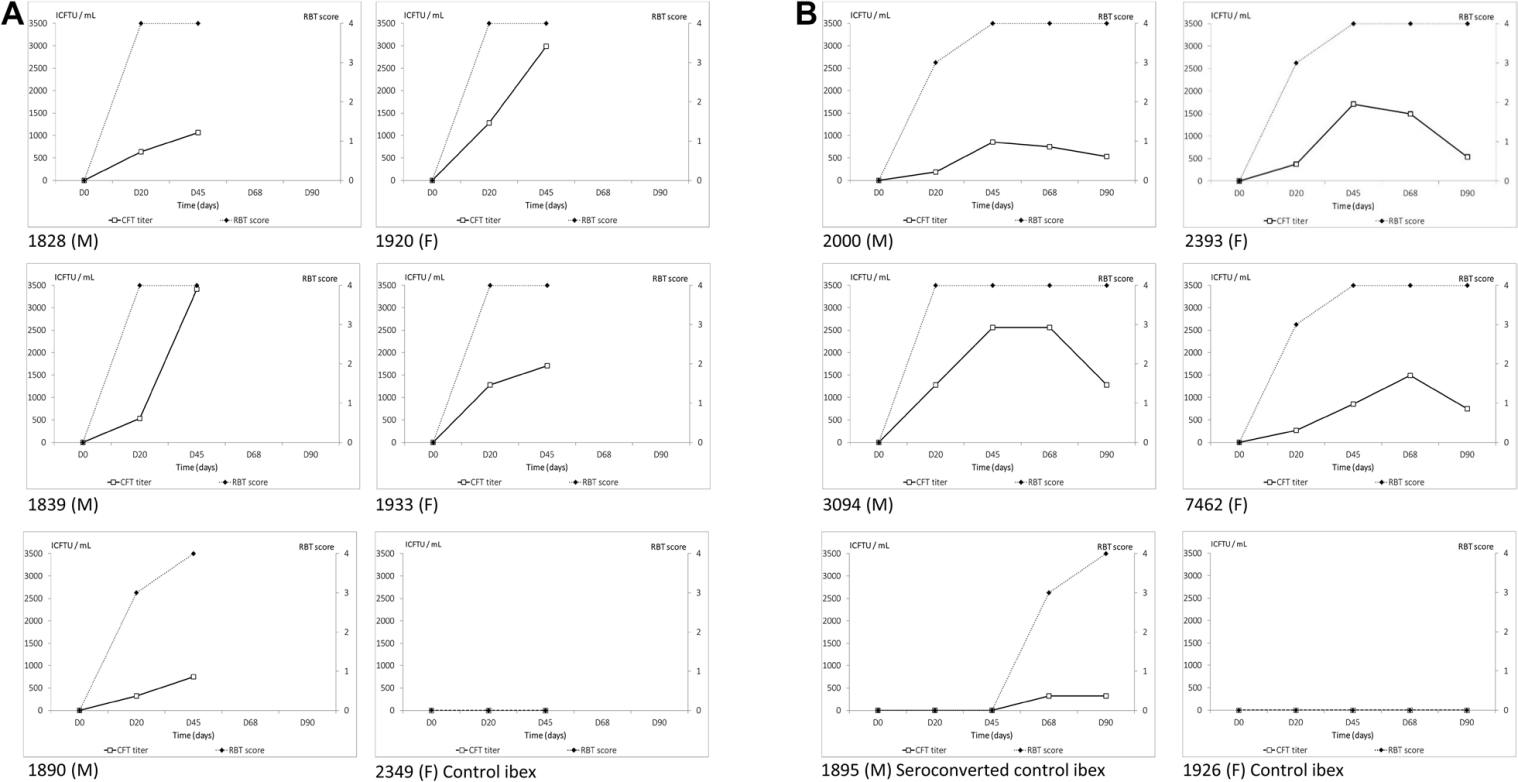
**Evolution of CFT titers (ICFTU/mL) and RBT scores in** ***Capra ibex***. **A** Vaccinated ibexes and control ibex (identified 2349) necropsied 45 days post-vaccination, and **B** vaccinated and control ibexes (identified 1926 and 1895) necropsied 90 days post-vaccination. Titers and scores were obtained on days 0, 20, 45, 68 and 90 after conjunctival vaccination on day 0 with *Brucella melitensis* Rev.1 strain, in both males (M) and females (F) (squares continuous lines for CFT titers and respectively diamonds in dotted lines for RBT score).

Changes in serological response after vaccination in both RBT and CFT are shown in Tables 3 and 4. Significant effects of day of sampling were observed for both CFT titers and RBT scores, with maximum values reached 20 and 45 days pv for RBT and CFT, respectively (3.6 ± 0.2 and 973 ± 187 ICFTU/mL). As expected, all animals showed seroconversion within 45 days pv [48] (Figures 4 and 5). However, the serological response (RBT score and CFT titers) was more intense and persistent in ibexes than in goats, with differences averaging 920 ± 257 ICFTU/mL and 1.5 ± 0.19 for RBT and CFT, respectively (*P* < 0.0001). The presence of bacteremia increased the antibody response observed with RBT. All ibexes remained seropositive throughout the experiment (with RBT- and CFT-positive results), whereas only one goat remained seropositive on day 90 pv as described in Ponsart et al. [48]).

**Table 3:** **Simultaneous tests for general linear hypotheses explaining RBT score and CFT titers (Lmer4 package, animal considered as random effect)**

Fixed effects	Level	CFT titers (ICFTU/mL)			RBT score		
		Estimate ± standard error	z value	Probability > |z|	Estimate ± standard error	z value	Probability > |z|
Intercept		516 ± 313	1.65	0.09	1.5 ± 0.3	6.0	< 0.0001
Day of sampling pv	0	–					
	20	449 ± 187	2.4	0.02	3.6 ± 0.2	19.4	< 0.0001
	45	973 ± 187	5.2	< 0.0001	3.6 ± 0.2	19.4	< 0.0001
	68	863.1 ± 239	3.6	0.0003	3.4 ± 0.2	14.7	< 0.0001
	90	460 ± 239	1.9	0.05	3.0 ± 0.2	13.1	< 0.0001
Species	Goat	–					
	Ibex	920 ± 257	3.6	0.0003	1.5 ± 0.19	7.7	< 0.0001
Bacteremia 20 days pv	No	–					
	Yes	128 ± 273	0.5	> 0.05	1.2 ± 0.2	5.9	< 0.0001

**Table 4:** **Mean CFT titers (mean titer ± SEM; CFT) and number of goats and ibexes positive in Rose Bengal tests (RBT) from 20 to 90** **days after conjunctival vaccination with** ***Brucella melitensis*** **Rev.1 strain (all samples were negative at D0 in both tests)**

Group	D20		D45		D68		D90	
	CF	RBT	CF	RBT	CF	RBT	CF	RBT
Necropsied on D45								
Vaccinated ibex (5)^a^	810 ± 397	5	1984 ± 1048	55				
Control ibex (1)	0	0	0	0				
Vaccinated goats (4)	322 ± 432	4	82 ± 61	4				
Control goats (0)	–	–	–	–				
Necropsied on D90								
Vaccinated ibex (4)	527 ± 440	4	1493 ± 708	4	1573 ± 646	4	773 ± 305	4
Control ibex (2)	0	0	0	0	0 vs. 160*	1^b^	0 vs. 160*	1^b^
Vaccinated goats (4)	45 ± 39	4	78 ± 63	4	25 ± 15	4	20 ± 35	3
Control goats (2)	0	0	0	0	0	0	0	0

^a^Number of animals per group.

^b^One male control ibex presented a seroconversion on day 68.

*Corresponding to the unvaccinated Ibex second control seroconversion.

### Kinetics and distribution of the *B. melitensis* Rev.1 vaccine

In total, 375 bacteriological samples were analyzed from the 22 animals that survived more than 20 days pv (i.e., 10 goats and 12 ibexes, 17 vaccinated and 5 unvaccinated). Of these 375 samples, 94 were found to be culture-positive [48]. All vaccinated animals exhibited at least one positive culture, all being positive at the time of necropsy in the head region, irrespective of the species or time post-vaccination (local initial multiplication). Furthermore, the contact ibex male was found to be infected by the Rev.1 strain (6 from 11 examined organs being culture-positive), while the four other contacts (one female and one male goat, two female ibexes) were found negative at the time of necropsy.

The proportion of culture-positive organs was studied among 50 sampling*individual events collected in vivo or at the time of necropsy among 17 vaccinated animals. Significant effects of time pv, of mode of sampling, and of species were observed, but there was no significant effect of animal gender (this was not retained in the best model according to the AIC model values). The probability of observing positive results was logically much lower during in vivo sampling compared to necropsy time (OR_in vivo/necropsy_ = 0.037, 95% CI [0.005; 0.289]). As expected, based on a previous study, this probability was also lower at day 90 pv compared to day 45 pv (OR_90 versus 45_ = 0.091, 95% CI [0.010; 0.860]). The proportion of culture-positive organs was higher in the ibexes compared to the domestic goats (OR_ibex/goat_ = 4.184, 95% CI [2.311; 7.574]). When focusing the analysis on the organs from the pelvic region, the species effect was again the most important factor (OR_ibex/goat_ = 7.222, 95% CI [2.192; 23.799]), and the mode of sampling was still significant (OR_in vivo/necropsy_ = 0.154, 95% CI [0.032; 0.733]), while the period had no significant effect.

In ibexes, average Rev.1 burdens in infected organs ranged from 1 to 300 on day 45 pv and from 1 to 100 at day 90 pv. In goats, average Rev.1 burdens ranged from 1 to 30 on day 45 pv and from 1 to 10 on day 90 pv. The bacterial burden (log-transformed) was studied for culture-positive organs examined at necropsy among 17 vaccinated individuals (either necropsied 45 or 90 days pv). Significant effects of time of necropsy (45 or 90 pv), of organ type, and species were observed, but there was no significant influence of animal gender. The bacterial burden was significantly lower in the head swabs (ocular or nasal) compared to any other organs, including genital and bladder swabs (*P*_Ex_head/Ex_genital_ = −4.876, 95% CI [−8.229; −1.523]). The bacterial burden was found to be about 10 times lower 90 days pv compared to 45 days pv (*P*_90 vs 45_ = −2.167^1^, 95% CI [−3.145; −1.188]). The bacterial burden of the culture-positive organs was also at least 10 times higher in ibexes compared to goats (*P*_ibex/goat_ = + 2.410 (see Footnote 1), 95% CI [+ 1.409; 3.411]). The interaction between the period and the species was not significant.

### Bacterial analyses in animal litter

None of the manure samples collected 68 days pv revealed the presence of *Brucella* (DNA detection and bacteria isolation). The efficiency and the sensitivity of both methods were comprised between 10^2^ and 10^4^ CFU/g using the *B. melitensis* reference strain (data not shown).

## Discussion

As expected during a successful Rev.1 vaccination, all vaccinated animals seroconverted and exhibited loco-regional Rev.1 strain multiplication. None of these vaccinated animals developed detectable lesions nor clinical signs attributable to the Rev.1 vaccine [5, 19, 49]. However, contrary to the experts’ expectations, our results revealed a highly contrasting vaccine outcome between the two species, which invalidated the hypothesis of comparable innocuousness of the Rev.1 vaccine between the Alpine ibex and the domestic goat.

### Rev.1 distribution and burden in Alpine ibex and domestic goat

The proportion of Rev.1-infected samples and the bacterial burdens observed in culture-positive organs were significantly higher in ibexes compared to goats. In goats, vaccination is likely to give rise to a bacteremia and a generalized infection [50]. Elberg and Meyer [51] showed that tissues of subcutaneous vaccinated goats have practically cleared themselves of Rev.1 vaccine by 14 weeks post-vaccination, a duration which is shortened through vaccination by the conjunctival route [51–53]. In this experiment, differences between species were the dominant factor, with negligible effects of individual factors such as gender. The proportion of infected organs, both at the whole individual level or focusing on the pelvic organs, decreased over time but remained moderate at 90 days pv in both species, with considerable individual variability. These results suggest a higher risk of Rev.1 shedding between day 20 and day 68 in ibex, but given the positive culture observed on day 90 pv, it is not possible to rule out potential persistence and shedding in the long term in vaccinated ibexes (or goats [19]). Sparse data have been published on vaccination of adult non-pregnant female deer. However, prolonged serological responses [54], as well as milk excretion, [22] have been reported, supporting the hypothesis of possible systemic infection. Two vaccinated male ibexes presented urogenital excretion 20, 45 or 68 days pv, which was not observed in the other vaccinated batches. As a logical consequence, the control male ibex sharing the same box seroconverted between 45 and 68 pv and exhibited a similar Rev.1 burden to its vaccinated fellows when necropsied 90 days pv. It has been previously shown that the persistence of Rev.1 in vaccinated goats on the conjunctiva, and in nasal secretion as well in the saliva is weakly detectable up to 15 days post vaccination [19]. This local scattering had no effect on sentinel goats (swabs and serology remained negative) indicating that there was no contamination by the vaccine strain (I. Jacques, personal communication). In Portugal, a study reported that sentinel animals remained negative in serology when placed with goats vaccinated by Rev.1 [55]. Given that no live bacteria were found in animal litter, and the low resistance of the Rev.1 vaccine to ultraviolet exposure, impacting its DNA repair mechanisms [56], one may presume that the sentinel male ibex infection was favored by the close contact with vaccinated males sharing the same box. The A1 housing used here is obviously not a comparable situation to the natural environment, where Rev.1 survival is certainly impaired by adverse environmental conditions (temperature, ultraviolet light, etc.). Nevertheless, the distribution of Rev.1 suggests a far lower ability of Alpine ibex to control Rev.1 multiplication, and a higher risk of Rev.1 shedding and further transmission to control individuals than in domestic species. Given this discrepancy, it is also very difficult to assess vaccine efficacy in Alpine ibex based on experiments done in domestic species; we may even hypothesize logical higher susceptibility of Alpine ibex to side effects such as abortions. Differences in vaccine effects in wild and domestic species have been observed, particularly in the American bison (*Bison bison*) and elk (*Cervus canadensis*) regarding *B. abortus* [57]. These American-native species were found to be more susceptible to abortion caused by attenuated live-vaccine than the domestic cow (*Bos taurus*) imported from Europe [26, 27, 30]. In the case of the Alpine ibex, we may hypothesize that its remote and sparse historical distribution [58] resulted in limited co-evolution with livestock pathogens including *Brucella*. In addition, intensively hunted Alpine ibex almost became extinct during the nineteenth century, and its recent restauration has been based on small animal numbers [59]. This historical background may have resulted in genetic bottleneck events and a consecutive decrease in immune capacity [60]. Further studies are clearly required to investigate these hypotheses and the mechanisms for species resistance regarding *Brucella* infection.

### Immune response

An important result of this experiment concerns the intensity of the highly contrasted immune reaction between the two species. This differentiated dynamic between goats and ibexes may reflect different immune response mechanisms between the two species. The *Brucellae* are intracellular bacteria that elicit both humoral and cell-mediated immune response. The S-LPS *Brucella* major antigen elicits a T-cell dependent immune response with IgG1 dominating. The switch between IgM and IgG occurs in less than 7 days in adults implying that the detection of the early humoral response mainly relies on IgG [61]. Goats vaccinated with the standard dose 1 × 10^9^ CFU by the conjunctival route showed a weak and short humoral response, negative after 4 months [49]. However, the antibody response is mostly more significant in adults than in immature animals [5, 61]. In this study, humoral response was more intense and prolonged in ibexes compared to goats on the basis of RBT and CFT, with persistent positive results in all ibexes until the end of the experiment. White cell counts exhibited a similar trend: an increase in the production of lymphocytes at the expense of neutrophils later, and that was more intense and lasting in ibexes compared to goats. Recently, a stronger pro-inflammatory response has been observed in *B. melitensis* 16 M-infected pregnant goats compared to Rev.1 at day 28 post-infection together with a greater rise in mononuclear numbers, thus highlighting impacts of strain attenuation on cell-mediated immune response in goats [50]. The development of the immune response to *Brucella* infection is not well documented in ibex, even though the high CFT titers observed in the Bargy area’s ibexes suggest a stronger and longer serological response in ibex naturally infected by *B. melitensis* biovar 3 compared to the domestic species infected by the same strain [47]. This study represents, to our knowledge, the first observation of longitudinal monitoring of seroconversion on this species following contact with a *B. melitensis* strain and over a duration of 3 months.

To conclude, the antibody dynamics support the evidence of different immune responses to the Rev.1 vaccine between the Alpine ibex and the domestic goat, with ibexes investing far more in humoral response than goats. In spite of this seemingly stronger immune response, the ibex seems much less effective than the goat in containing the multiplication of this intracellular bacterial strain. Comparable strong antibody responses have previously been reported in elk vaccinated with RB51, together with prolonged bacteremia and slower detectable proliferative responses in PBMC when compared to responses in cattle or bison [62, 63]. Protection against *Brucella,* as an intracellular pathogen, is believed to be mainly associated with Th1-type cell response, and the subsequent activation of cytotoxic T cells, NK cells, and macrophages [64]. In comparison, cytokines associated with a Th2-type response can stimulate antibody responses but can also have negative regulatory effects on Th1-type responses [65]. In the elk species, despite robust antibody responses to *Brucella*, the Th1–Th2 paradigm was the main hypothesis to explain the lack of cellular immune responses and associated reductions in vaccine protection [29, 63]. Our results lead to similar hypotheses in the ibex species. This might correspond to a “naïve” immune response in wild species, possibly because they did not co-evolve with *Brucella*, unlike domestic ruminant species, and/or to an intrinsic lower immune capacity regarding this pathogen. Further studies are required to better understand immune responses in wild and domestic ruminant species regarding *Brucella*, and their involvement in disease development and shedding outcomes.

In conclusion, tissue localization, shedding of bacteria, and humoral immune responses differ between Alpine ibexes and goats after conjunctival vaccination with *B. melitensis* Rev.1 strain. Ibex expressed more intense and prolonged humoral immune response than the domestic goat, whereas the distribution and organ burdens of the vaccine strain were at least 10 times higher in ibex, particularly in the urogenital organs. Two out of five vaccinated male ibexes shed Rev.1 during the experiment, which resulted in the transmission of Rev.1 to the control male and its seroconversion. Alpine ibexes showed a lower capacity to contain the Rev.1 live-vaccine than the domestic species, and thus represent a higher risk of vaccine strain shedding. The practical constraints associated with the use of the vaccine *in natura* i.e., vaccination only possible in captured ibexes in the spring, including pregnant females, are likely to worsen the excretion of vaccine strain spread, with potentially disruptive effects on the monitoring and management of this wild reservoir. This study focused on the vaccine’s innocuousness and not on its efficacy or field deployment; therefore, our conclusion is not definitive concerning vaccine use *in natura*, but could contribute to ongoing risk assessment and research addressing the efficacy of disease management strategies.

## Abbreviations

BTV: Blue Tongue Virus (Ovine Catarrhal Fever)
BVD: bovine viral diarrhea
CAEV: caprine arthritis encephalitis virus
CFT: complement fixation test
ELISA: Enzyme-Linked ImmunoSorbent Assay
PBS: phosphate buffered saline
PBMC: peripheral blood mononuclear cell
pv: post-vaccination
QF: Q fever
RBT: Rose Bengal test

## Footnotes

1 The data have been log-transformed; please consider the parameter on the log-scale.
